# Combining transcriptome and metabolome analysis to understand the response of sorghum to *Melanaphis sacchari*

**DOI:** 10.1186/s12870-024-05229-8

**Published:** 2024-06-11

**Authors:** Xin-Rui Zhao, Dong-Ting Zhao, Ling-Yu Zhang, Jin-Hua Chang, Jiang-Hui Cui

**Affiliations:** 1 College of Agronomy, Hebei Agricultural University, Baoding, 071000 China; 2 North China Key Laboratory for Crop Germplasm Resources of Education Ministry, Baoding, 071000 China

**Keywords:** Sorghum aphid, Sorghum, Transcriptome, Metabolome, Flavonoid metabolic pathway

## Abstract

**Background:**

The sorghum aphid *Melanaphis sacchari* (Zehntner) (Homoptera: Aphididae) is an important insect in the late growth phase of sorghum (*Sorghum bicolor* L.). However, the mechanisms of sorghum response to aphid infestation are unclear.

**Results:**

In this paper, the mechanisms of aphid resistance in different types of sorghum varieties were revealed by studying the epidermal cell structure and performing a transcriptome and metabolome association analysis of aphid-resistant and aphid-susceptible varieties. The epidermal cell results showed that the resistance of sorghum to aphids was positively correlated with epidermal cell regularity and negatively correlated with the intercellular space and leaf thickness. Transcriptome and metabolomic analyses showed that differentially expressed genes in the resistant variety HN16 and susceptible variety BTX623 were mainly enriched in the flavonoid biosynthesis pathway and differentially expressed metabolites were mainly related to isoflavonoid biosynthesis and flavonoid biosynthesis. The q-PCR results of key genes were consistent with the transcriptome expression results. Meanwhile, the metabolome test results showed that after aphidinfestation, naringenin and genistein were significantly upregulated in the aphid-resistant variety HN16 and aphid-susceptible variety BTX623 while luteolin was only significantly upregulated in BTX623. These results show that naringenin, genistein, and luteolin play important roles in plant resistance to aphid infestation. The results of exogenous spraying tests showed that a 1‰ concentration of naringenin and genistein is optimal for improving sorghum resistance to aphid feeding.

**Conclusions:**

In summary, the physical properties of the sorghum leaf structure related to aphid resistance were studied to provide a reference for the breeding of aphid-resistant varieties. The flavonoid biosynthesis pathway plays an important role in the response of sorghum aphids and represents an important basis for the biological control of these pests. The results of the spraying experiment provide insights for developing anti-aphid substances in the future.

**Supplementary Information:**

The online version contains supplementary material available at 10.1186/s12870-024-05229-8.

## Introduction

Sorghum (*Sorghum bicolor* L.) represents the fifth most grown crop worldwide after maize, wheat, and rice [1–3] and is a C4 crop with high nitrogen and water use efficiency [4]. It has a strong environmental adaptability and wide distribution and is extensively grown in arid and semi-arid regions worldwide, thus playing a vital role in the world’s agroecosystems [4].The sorghum aphid (*Melanaphis sacchari* (*Zehntner*) (*Homoptera: Aphididae*) is a major pest of sorghum that seriously harms the yield and quality of sorghum under field conditions [5]. Sorghum aphids occurr on sugarcane and sorghum in India, China, South Africa, Japan, and the United States, mainly during late sorghum growth [6–8]. Sorghum aphids are widely distributed in sorghum and sugarcane growing areas [8], and their population growth is influenced by temperature and rainfall patterns [9], with populations increasing rapidly in warm and dry climates [8]. Moreover, the timing and severity of infestation vary with location and year [10–12]. Sorghum aphids cause increased water stress in plants as they feed on the underside of sorghum leaves and stalks and suck plant sap. This feeding process causes the direct loss of plant nutrients and sugars. [6, 7, 13]. If sorghum is infested with aphids during panicle initiation and flowering, it can lead to delayed plant development, reduction in the number of spikes, and reduction in the accumulation of photosynthates, which overall reduces sorghum yield. Sorghum aphids also important pests of forage sorghum because they secrete large amounts of honeydew during host survival, which can cause sorghum leaves to stick together. Moreover, the excessive accumulation of honeydew can lead to mechanical problems during cutting and baling and reduce harvesting efficiency and quality because of the associated growth of mold [14]. A cosmopolitan pest, the sorghum aphid has high dispersal and reproductive potential and can rapidly increase in number. A single female can produce up to 75 nymphs in 12 days [6], whereas 50 sorghum aphids can produce up to 500 nymphs within a week [6]. This significantly increases the risk of plant infestation. Once infested, sorghum aphids can grow exponentially and reach 30,000 aphids on a single plant [8]. This can cause direct as well as indirect economic losses. In large-scale sorghum cultivation with many sensitive varieties, sorghum aphids remain the most damaging pests. In this context, the promotion of biological control of aphids has gradually increased to improve the environmental sustainability of sorghum. Chemical spraying is the main method of sorghum aphid control; however, this method not only pollutes the environment and leads to insect resistance but also kills other beneficial insects, such as predators, parasites, and pollinators. Therefore, alternative methods of effective aphid control are required.

During the co-evolution of plants and insects, both have acquired strategies to avoid each other’s defense systems [1, 2]. The interaction between plants and insects has led to the development of complex plant defense systems that can recognize infectious molecules or signals in damaged cells and activate the plant’s immune response to herbivorous insects [1, 2, 14–16]. Plants respond to herbivore attacks through a complex and dynamic defense system that includes structural barriers, toxic chemicals, and natural enemies of target pests [15, 17, 18]. Plant-induced responses are important components of agricultural pest control and have been widely used to regulate populations of herbivorous insects [6, 19, 20]. The substances that induce defense are primarily secondary metabolites, including phenolic and flavonoid compounds. Secondary metabolites are compounds that do not affect normal plant growth and development but reduce the palatability of the plant tissues in which they are produced [2, 21]. Defensive (secondary) metabolites can be constitutive, stored in an inactive form, or induced upon insect or microbial attack. Secondary metabolites protect plants from different stresses and enhance their adaptive capacity [22, 23]. Aphid infestation of aphid-sensitive sorghum has resulted in the altered expression of functional genes involved in cell wall modification, photosynthesis, and phytohormone biosynthesis [24]. Dhurrin is a cyanogenic glycoside found in sorghum that likely plays an important role in plant–herbivore defense [25, 26]. Jasmonic acid mediated responses may play a key role in promoting herbivore resistance [25, 26]. However, the mechanisms underlying aphid resistance in sorghum require further investigation.

To further elucidate the aphid resistance mechanisms of sorghum, aphid stress experiments were conducted on the aphid-susceptible variety BTX623 and aphid-resistant variety HN16, which are widely grown in China. The microstructures of the leaves of aphid-susceptible and aphid-resistant varieties of sorghum were observed, and the transcriptome and metabolic changes in their leaves before and after aphid infestation were analyzed. Candidate genes, metabolites, and key pathways were then identified. Real-time quantitative polymerase chain reaction (qRT-PCR) was used to validate candidate differentially expressed genes (DEGs). We conducted exogenous spraying experiments to determine whether metabolites affect aphid resistance in sorghum, identify efficient aphid control methods, and provide a reference for the development of aphid-resistant substances in sorghum.

## Materials and methods

### Microstructure observation of different resistant varieties

Aphid-resistant varieties HN16, Silimei, and BTX428 and aphid-sensitive varieties BTX623, QianSan, and 3197B were selected for sampling at the heading stage. This was followed by fixation with FAA fixation, paraffin embedding, sectioning, and staining saffron solid green to observe the epidermal arrangement of different aphid-resistant varieties under a microscope (Nikon, Japan, Nikon Eclipse E100). The epidermal cell parameters and relative thickness were determined using CaseViewer software.

### Transcriptome determination

The representative high-resistance variety HN16 and the sensitive variety BTX623 were selected for the aphid infestation test. At the sorghum heading stage, aphids were lightly brushed on the lower surface of the 4th and 5th leaf with a brush. At 0, 1, 2, 3, and 4 days, aphid-infested parts of the leaves were collected to form mixed samples, and 10 samples were sequenced using a transcriptome. According to the sampling days, HN16 was numbered CKh (0d), Th1 (1d), Th2 (2d), Th3 (3d), Th4 (4d); and BTX623 was numbered CKb (0d), Tb1 (1d), Tb2 (2d), Tb3 (3d), Tb4 (4d) (Additional file 1). RNA concentration and purity were measured using a NanoDrop 2000 spectrophotometer (Thermo Fisher Scientific). RNA integrity was assessed using an RNA Nano 6000 Assay Kit on an Agilent Bioanalyzer 2100 system (Agilent Technologies, CA, USA) [27]. A total amount of 1 μg RNA per sample was used as input material for the RNA sample preparation [27]. Sequencing libraries were generated using the NEBNext UltraTM RNA Library Prep Kit for Illumina (NEB, USA), following the manufacturer’s recommendations. Index codes were added to attribute sequences for each sample. Hisat2 tools soft were used to map with reference genome [28]. The reference genome was downloaded from https://www.ncbi.nlm.nih.gov/genome/term=sorghum. Differential expression analysis of the two samples was performed using edgeR. FDR < 0.01 and fold change ≥ 2 were set as the thresholds for significantly differential expression [29].

### Metabolome determination

Based on the results of the transcriptome assessment, a metabolome assessment was performed on HN16 and BTX623 cells at 0 and 3 days afterinfestation, respectively. Each treatment was repeated three times for a total of 12 samples. The samples and transcriptome materials belonged to different biological repeats of the same batch. Sample extraction, chromatomass spectrometry collection, and metabolite characterization and quantification were conducted by NetWare (Wuhan, China) in accordance with standard procedures and previous studies [30]. The software Analyst 1.6.3 was used to process the mass spectrum data. Significantly regulated metabolites between groups were determined by VIP ≥ 1 and absolute log2FC (fold change) ≥ 1 [29].

### Quantitative real-time PCR

HN16 and BTX623 plants infested with aphids for 0 and 3 days were used for quantitative real-time PCR (qPCR). Transcriptome sequencing was performed using the same batch of biological replicates. RNA was extracted with reference to the Eastep® Super Total RNA Extraction Kit (SHANGHAI PROMEGA) kit [31]. cDNA synthesis was performed using HiFiScript gDNA Removal RT MasterMix (CWBIO) and cDNA was synthesized with reference to the AugeGreenTM qPCR Master Mix (US EVERBRIGHT, UE) kit for the step-by-step method using cDNA as the template and actin as the internal reference gene [27]. The qRT-PCR system was configured with 20ul, and the relative expression of genes was calculated using the 2-ΔΔCt method with an ABI7500 PCR instrument from Applied Biosystems for real-time quantification of fluorescence [32]. qRT-PCR was performed in triplicate for each gene. The qPCR temperature cycler was set to 95 °C for 2 min, 95 °C for 5 s, 60 °C for 30 s, and 45 cycles. Primer design was performed using Primer 5 software (Additional file 2).

### Exogenous spraying test

The test varieties were the resistant variety HN16 and susceptible variety BTX623, while qiansan naringenin, luteolin, and henistein (95% purity, purchased on August 5, 2022, San Land, USA) were chosen as metabolites. Aphids were extracted from other plants that had been infested earlier in other fields.The field spraying concentrations were set to 0, 0.2‰, and 0.1‰ (Additional file 3). The metabolites were sprayed on the front and back of the sorghum leaves, and three plants were sprayed in each treatment, with each treatment replicated three times.

## Results

### Microstructure of *sorghum* leaves of different resistant varieties

**Fig. 1:**
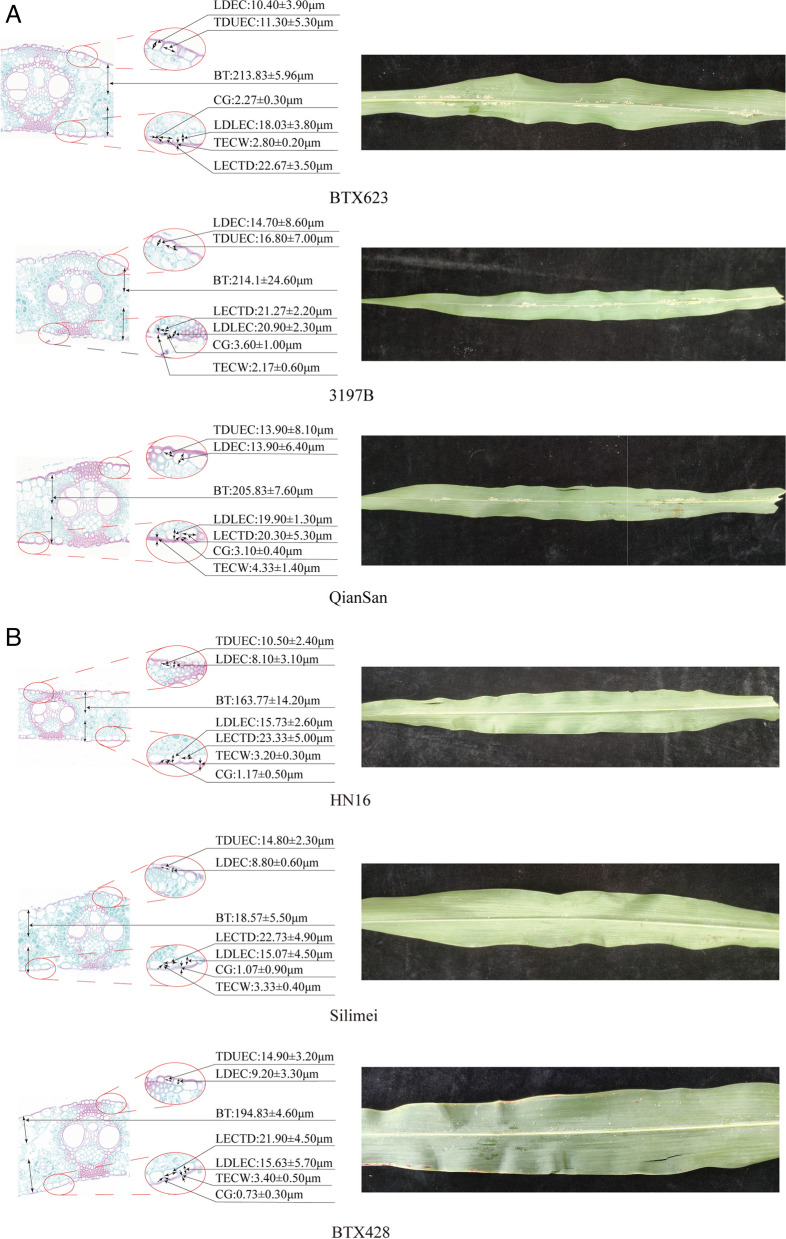
Microstructure of sorghum leaves and changes in aphid population after three days of inoculation. **A** Microstructure of leaves of sensitive sorghum varieties and their aphid population after three days of inoculation with aphids. **B** Microstructure of leaves of resistant sorghum varieties and their aphid population after three days of inoculation with aphids. Note: LDEC, longitudinal diameter of upper epidermal cells; TDUEC, transverse diameter of upper epidermal cells; LDLEC, Longitudinal diameter of lower epidermal cell; LECTD, lower epidermal cell transverse diameter; LEC L/T: lower epidermal cells longitudinal/transverse; CG, cell gap; BT, blade thickness; TECW, thickness of epidermal cell wall

The epidermal structure of the leaves differed significantly between the aphid-susceptible varieties (Fig. 1A, Additional file 4: L4-6) and aphid-resistant varieties (Fig. 1B, Additional file 4: L7-9). The epidermal cells of the aphid-resistant varieties (HN16, Silimei, and BTX428) were neatly and tightly arrange while those of the aphid-sensitive varieties (BTX623, QianSan, and 3197 B) were irregular in shape, uneven in cell size, and unevenly arranged, and had large cell gaps. Evaluation of the epidermal cell characteristics and leaf thickness and analysis of significant differences showed that bose inand aphid-resistant varieties and aphid-susceptible varieties (Additional file 4), the longitudinal and transverse diameters of upper epidermal cells differed insignificantly. In aphid-resistant varieties the longitudinal and transverse diameters of lower epidermal cells differed more and were closer to a rectangular shape compared with that in aphid-susceptible varieties, which were closer to a round shape. In aphid-resistant varieties, the cell gap was smaller and the cells were more tightly arranged, in aphid-susceptible varieties, which presented a larger cell gap and loosely arranged cells, the differences were significant. The difference in blade thickness was highly significant, with aphid-resistant varieties presenting thinner blades than aphid-susceptible varieties. In summary, the resistance of sorghum to aphids was positively correlated with the regularity of epidermal cells and negatively correlated with intercellular space and leaf thickness.

### Transcriptomic analysis of *sorghum* leaves

After sequencing quality control, a total of 78.68 GB of clean data were obtained. The clean data from each sample reached 7.12 GB, and the distribution of Q30 bases in each sample ranged from 94.67% to 94.95%. The GC content ranged from 53.09% to 55.06%. Clean reads were subjected to sequence alignment with the specified reference genome, with alignment efficiencies ranging from 90.17% to 94.46% (Additional file 5).

**Fig. 2:**
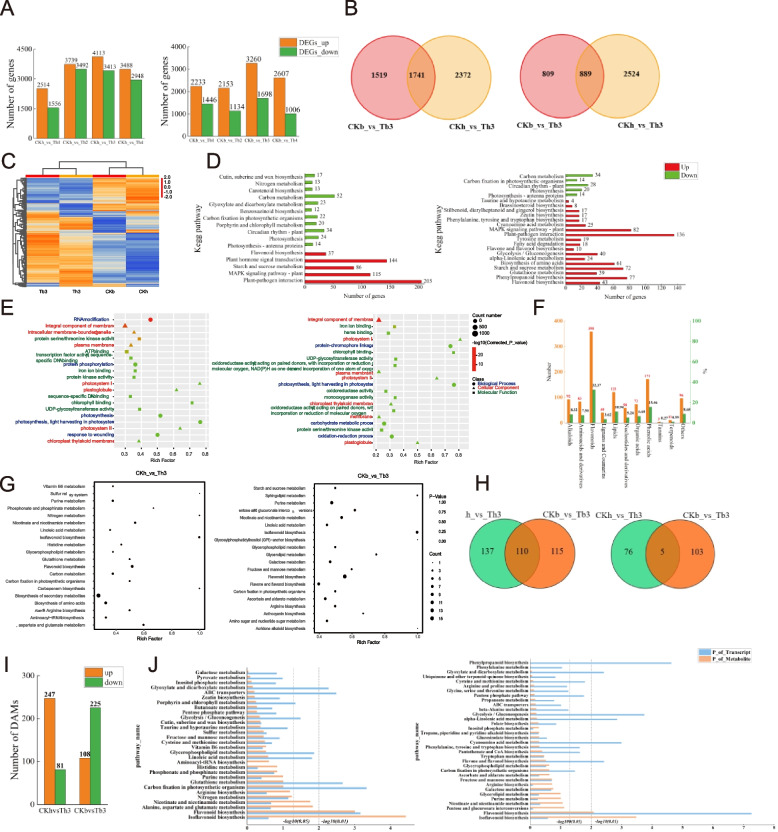
Transcriptome and metabolome analysis of different comparison groups after sorghum aphid infestation. **A** Number of DEGs in sorghum infested with aphids at different days after infestation compared to the control. **B** DEG Veen plot of different varieties of sorghum infested with aphids compared to the control. **C** Heat map of expression clustering of DEGs for each treatment. **D** DEG GO enrichment map after sorghum infestation by aphids compared to the control. **E** Histogram of DEG KEGG enrichment after sorghum infestation by aphids compared to the control. **F** Taxonomic map of metabolites detected through metabolomics of sorghum leaves. **G** KEGG enrichment map of DAMs after sorghum infestation by aphids compared to the control. **H** DAMs Veen diagram of different varieties of sorghum infested with aphids compared to the control. **I** Number of DAMs after sorghum infestation by aphids compared to the control. **J** Sorghum infestation by aphids after joint analysis with control compared to KEGG. Note: In panels **A**, **D**, **E**, **G**, and **J**, both left plots are HN16 and both right plots are BTX623; and in panels **B** and **H**, both left plots are upregulated and both right plots are downregulated

A total of 3,462 new genes were identified and 1,572 genes were annotated throughout the project. HN16 and BTX623 produced the highest number of differentially expressed genes three days after aphid infestation (Fig. 2A). Therefore, we further analyzed the differentially expressed genes in HN16 and BTX623 infested with aphids for 0 and 3 days. Among them, 7526 differential genes were identified in HN16, including 4113 upregulated genes, which were mainly enriched in the plant–pathogen interaction and MAPK signaling pathway, that is, plant, starch, and sucrose metabolism, and 3413 downregulated genes, which were mainly enriched in photosynthesis, that is, antenna proteins, photosynthesis, circadian rhythm, plant, and other pathways. A total of 4958 differential genes were identified in BTX623, including 3260 upregulated genes, mainly enriched in flavonoid biosynthesis, phenylpropanoid biosynthesis, and glutathione metabolism pathways. There were 1698 downregulated genes, mainly in photosynthesis-antenna proteins, photosynthesis, circadian rhythm, plant, and other pathways (Additional file 6, Sheets 1–2).

In the comparison with the Venn diagram of CKh vs. Th3 and CKb vs. Tb3 (Fig. 2B), 1741 differential genes were co-upregulated and 889 differential genes were co-downregulated. There were 1519 and 809 DEGs that were only downregulated in BTX623, and 2372 and 2524 DEGs that were only downregulated in HN16. The results showed that some genes changed at the transcriptional level after sorghum leaves were invaded by aphids. The clustering heat map shows the changes in the expression of each differential gene (Fig. 2C). GO analysis showed that the DEGs were enriched in three major functional categories, molecular function (MF), cellular component (CC), and biological process (BP) (Additional file 7, Sheets 1–6). DEGs in CKh compared with Th3 were enriched in all three GO classes. In the CC class, the integral components of the membrane, intracellular membrane-bounded organelles, and plasma membranes were the most enriched. In the BP and MF classes, RNA modification and protein serine/threonine kinase activity were the most abundant. DEGs of CKb relative to Tb3 were most abundant in CC and MF. The integral component of the membrane was most abundant in the CC class, and iron-ion binding was most abundant in the MF class (Fig. 2D).

To further analyze the biological functions of the DEGs, we performed KEGG enrichment analysis (Fig. 2E). Differential genes were upregulated in the comparison between CKh and Th3 and mainly related to plant–pathogen interactions, MAPK signaling pathway-plants, and flavonoid biosynthesis. In the comparison between CKb and Tb3, the upregulated genes were associated with flavonoid biosynthesis, phenylpropanoid biosynthesis, and glutathione metabolism. The downregulated genes in the two comparison groups were related to photosynthesis–antenna proteins, photosynthesis, and circadian rhythm-plants. These results show that aphid infestation inhibited the expression of genes related to photosynthesis antenna proteins, circadian rhythm, and decreased photosynthesis in sorghum. However, porphyrin and chlorophyll metabolism, benzoxazinoid biosynthesis, glyoxylate and dicarboxylate metabolism, and carotenoid biosynthesis were only downregulated in HN16 after aphid infestation. Plant hormone signal transduction was only upregulated in HN16 but not in aphid-infested BTX623. The upregulated metabolic pathways specific to BTX623 were phenylpropanoid biosynthesis, glutathione metabolism, biosynthesis of amino acids, and alpha-linolenic acid metabolism. This suggests that there may be differences in the mechanisms of different resistant varieties in response to aphid stress.

### Metabolomic analysis of *sorghum* leaves

Using the UPLC-MS/MS detection platform, we detected 1106 metabolites. Subsequently, all the metabolites were classified into 11 major groups, the most diverse of which comprised 358 flavonoids, 171 phenolic acids, and 121 lipids (Fig. 2F). Many differential metabolites were identified in different comparisons, including 247 upregulated and 81 downregulated metabolites in the comparison of CKh with Th3, and 108 upregulated and 225 downregulated metabolites in the comparison of CKb with Tb3 (Fig. 2I, Additional file 8, Sheet 1).

The Venn diagram showed that there were 110 common upregulated metabolites in the comparison of CKh vs. Th3 and CKb vs. Tb3. Among these, flavonoids were the most abundant, comprising 74 types, accounting for 67.27% of the total upregulated metabolites. This was followed by lipids, comprising 14 types, accounting for 12.73% of the total upregulated metabolites, five types of common downward adjustment, one type of amino acid and their derivatives, one type of organic acid, two types of nucleotides and their derivatives, and one type of phenolic acid (Fig. 2h, Additional file 8 Sheet 1). The expression of the three flavonoids 2'-hydroxygenistein, naringenin, and 6-hydroxyluteolin was upregulated after aphid infestation of HN16 and BTX623. The expression of a nucleotide and its derivative, Inosine 5'-monophosphate, was downregulated after aphid infestation. In addition, 45 differential metabolites were regulated based on a comparison of CKh vs. Th3 and CKb vs. Tb3 (Additional file 8).

To further highlight the pre-and post-infestation profiles of the two sorghum varieties, we created a clustering heat map for the 160 DAMs mentioned above (Additional file 9). These metabolites were divided into nine species, that is, flavonoids, nucleotides and derivatives, and lipids. Most of the differential metabolites increased after BTX623 was infested with sorghum aphid compared to HN16 infestation, with a significant trend in the expression of flavonoid substances in BTX623 and HN16. For example, pme2960 Naringenin chalcone and Lmmp004504 2'-hydroxygenistein showed a significantly higher upward trend in expression in BTX623 than in HN16. Meanwhile, pmp000587 luteolin-7-*O*-(6''-malonyl) glucoside showed a significant upward trend in expression in HN16. In summary, sorghum aphid infestation had a significant effect on BTX623 and a moderate effect on HN16, particularly on flavonoid metabolites.

Based on the KEGG annotation, differential metabolites in different comparisons were enriched in many pathways, including flavonoid, amino acid, and secondary metabolite biosynthesis. Among the top 20 KEGG-enriched pathways in the different comparisons, those in the CKh vs. Th3 and CKb vs. Tb3 comparisons were the most significantly enriched in isoflavonoid biosynthesis, followed by flavonoid biosynthesis (Fig. 2G). This shows that metabolites enriched in flavonoid and isoflavonoid biosynthesis may play a role in direct or indirect defense against aphids.

### Association analysis of metabolome and transcriptome

**Fig. 3:**
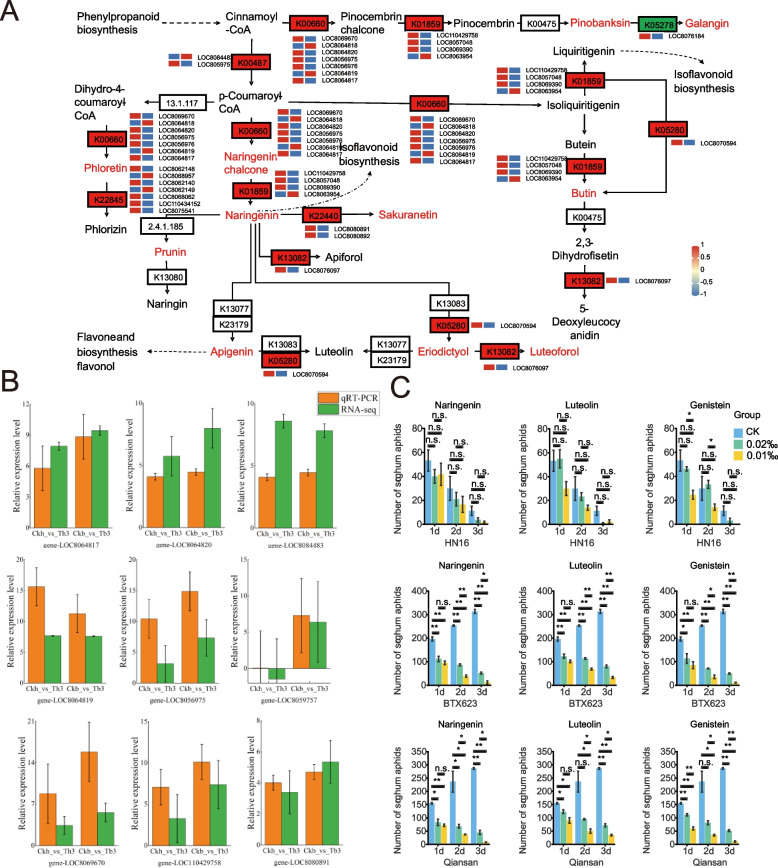
Flavonoid metabolic pathway and exogenous spraying (**A**) Flavonoid metabolic pathway, key genes, and key metabolites in red, DEG expression heat map CKb vs. Tb3 on the left, and CKh vs. Th3 on the right. **B** Transcriptome of different genes with real-time quantitative bar graphs. **C** Statistical plot of the aphid population after exogenous spraying of metabolites on sorghum leaves; * indicates a significant difference (*p* < 0.05) and n.s. indicates no significant difference. Note: The copyright license has been applied for

To more effectively visualize the co-enrichment of differential genes and metabolites, transcriptomic metabolomic KEGG enrichment analysis was performed (*p* < 0.05), and the pathway of the top 20 differential genes and metabolites for each group were enriched(Fig. 2J).The two varieties were enriched in some common functional pathways, such as flavonoid biosynthesis and isoflavonoid biosynthesis. DEGs and DAMs were enriched in both varieties, suggesting that these metabolic pathways may play a crucial role in sorghum resistance against aphids(Fig. 3A). Therefore, we analyzed the main DEGs and DAMs involved in these pathways. By comparing CKb vs. Tb3 and CKh vs. Th3, we identified 40 flavonoid biosynthesis-related genes, including chalcone synthase (CHS), isoflavone 3'-hydroxylase (I3’H), cytochrome P450 CYP73A100(P450), hydroxycinnamoyltransferase (HCT), and isoflavone 2'-hydroxylase (I2’H) (Additional file 10, Sheet 1). Among these 40 genes, the expression of 36 genes was significantly upregulated while that of 4 genes was significantly downregulated between CKb and Tb3. In addition, the expression of 33 genes was significantly upregulated while that of 7 genes was significantly downregulated between CKh and Th3. In the metabolomic analysis, flavonoid biosynthesis involved 18 differential metabolites such as naringenin, genistein, and apigenin. These metabolites were significantly upregulated in BTX623 and HN16 (Additional file 10, Sheet 2).

### qRT-PCR

To validate the differential expression results obtained from the transcriptome analysis, we examined the relative expression levels of the nine selected regulatory genes using qRT-PCR. These genes are involved in flavonoid biosynthesis, including eugenol O-methyltransferase-like, C4H, CHS, and cytochrome P450 CYP73A100. The expression patterns of DEGs obtained using RNA-seq and qRT-PCR were highly consistent, highlighting the reliability of the RNA-seq results (Fig. 3B).

### Validation of flavonoid metabolite spraying

To verify whether the screened metabolites would affect aphid resistance in sorghum, three sorghum varieties with different aphid resistance were selected for exogenous spraying experiments, they were the aphid-resistant varieties HN16 and aphid-sensitive varieties BTX623 and Qiansan. Among the metabolites involved in the flavonoid biosynthesis pathways, three metabolites, that is, naringenin, luteolin, and genistein, were used for foliar spraying using more mature extraction techniques, more widespread plant sources, and lower application costs. The results showed that all three metabolites had a greater control effect on aphids. Aphid reduction in the different resistant varieties was higher than those without the metabolites sprayed (Fig. 3C). However, the control effect of spraying different concentrations of metabolic substances was different, and the different treatments showed that the number of aphids sprayed with 0.2‰ concentration was significantly higher than that of 0.1‰ concentration. This indicates that spraying 0.1‰ concentration of metabolic substances could play a more effective role in controlling the aphid population. Three days after spraying, genistein at 0.1‰ concentration was more effective against aphids in BTX623, followed by naringenin at 0.1‰ concentration; naringenin at 0.1‰ concentration was more effective against aphids in QianSan, followed by genistein at 0.1‰ concentration, at HN16, the aphid population in all groups showed a decreasing trend, but spraying with 1‰ genistein showed the fastest decreasing trend, followed by spraying with 1‰ naringenin. This shows that spraying 1‰ concentration naringenin and 1‰ concentration genistein was more effective than spraying luteolin for aphid control.

## Discussion

Physical defenses are the first line of defense against herbivorous insects and are mainly morphological and anatomical features that can directly prevent feeding by herbivorous insects and provide plants with a fitness advantage [33]. This includes prominent protrusions on the plant and microscopic changes in cell wall thickness from lignification and sub lignification [33–35]. The present study has shown that sorghum aphid resistance was positively correlated with epidermal cell arrangement tightness and negatively correlated with leaf thickness, which is in line with the results of Chang et al. [36]. In contrast, Luo et al. [37]. showed that cotton leaf thickness was not related to resistance to the *Apolygus lucorum*. Zhong et al. [38]showed that aphid resistance in tea trees was positively correlated with leaf thickness. These results indicate the differential activity of aphids in different crops.

Transcriptomic and metabolomic analyses have become common tools for evaluating interactions between plants and herbivorous insects [39]. In this study, we used a combined transcriptomic and metabolomic approach to comparatively analyze the genetic and metabolic changes involved in different genotypes of sorghum subjected to aphid infestation. This has increased our understanding of the potential mechanisms by which sorghum responds to aphid infestation. Studies have shown that in sorghum, rice and maize plants infested by sorghum aphids, rice stem borers (*Chilo suppressalis*), and corn aphids, the number of upregulated DEGs is higher than the number of downregulated DEG [40–42], indicating that insect feeding triggers host transcriptome recombination. Similarly, our DEG analysis showed that there were more upregulated DEGs than downregulated DEGs in response to aphid infestation. However, Wang et al. [43] showed that more DEGs were downregulated than upregulated when rice was infested with brown flies. Li et al. [44] showed that the number of upregulated and downregulated DEGs was similar when cotton was infested with sooty flies. These diverse results may be related to differences in aphid resistance between sorghum and other plants, differences in feeding between sorghum aphids and other insects, differences in the number of leaves infested by sorghum aphids compared to the number of plants stimulated by other herbivorous insects, or differences in the techniques used to detect gene expression.

The infestation of sorghum aphids can initiate a direct defense response of sorghum, induce flavonoid biosynthesis gene expression, and promote flavonoid production. The flavonoid biosynthesis is the most abundant pathway in sorghum after aphid infestation. In this pathway, aphid feeding increased gene expression of enzymes involved in sorghum flavonoid biosynthesis, including CHS, I3 'H, and HCT. This is consistent with previous research on transferase activities upregulated in later stages of infestation [45]. Activation of signal transduction pathways after an insect attack leads to concomitant changes in plant secondary metabolism [46]. Metabolomic analysis showed that the generalized upregulation of flavonoid biosynthesis and isoflavonoid biosynthesis under insect feeding seems to be a common strategy for sorghum to resist aphid feeding. Some metabolic substances in the pathway, such as genistein, naringenin, and naringenin chalcones, were significantly increased in abundance. This result further suggests that flavonoids may play a key role in their defense against aphids.

In a previous study by our experimental group, the sorghum aphid resistance gene RMES1 was localized and cloned in HN16 [47]. The results showed that sorghum material carrying the RMES1 gene was significantly more resistant to aphids than material that did not carry the RMES1 gene or material that had a mutation in the RMES1 gene. For example, HN16, which carries the RMES1 gene, is more resistant to aphids than BTX623, which does not carry the RMES1 gene. The differential genes screened in this study were mainly enriched in the flavonoid bioconjugate completion pathway. This is a major class of phytochemicals accounting for 5–10% of the known plant secondary metabolites [48]. Most of these have strong antioxidant activity and protect plants from insect pests by influencing the behavior, growth, and development of insects [49]. Depending on the dose applied, this group of metabolites can have different effect on the insect's feeding behavior, survival, and development [50–52]. The results have shown that naringenin, genistein, and luteolin reduced the number of aphids in sensitive plants while naringenin and genistein significantly increased aphid resistance in sensitive sorghum. This is consistent with the results of previous studies that demonstrated that naringenin, luteolin, and its derivatives have antibacterial and insecticidal activities [53–59]. The reduction in aphid numbers was significantly higher at 0.1‰ than at the other two concentrations. The results show that aphid feeding resistance does not necessarily increase with a higher concentration of metabolites. Previous studies have confirmed that exogenous JA has dual effects on aphid resistance of sorghum and will promote the growth and reproduction of aphids when the concentration exceeds the sensitivity of aphids [60]. The flavotoxin compounds extracted from *Fusarium petiolate* showed strong insecticidal activity at low concentrations [61]. Therefore, we concluded that sprayed flavonoids have dual effects on sorghum's resistance to aphid feeding. The reason for this phenomenon may be that the plant absorbs excessive flavonoids, resulting in excessive production and accumulation of ROS, which destroys the balance between oxidants and antioxidants and affects the ability of sorghum to resist aphids [62].

## Conclusion

We observed the microstructure of the leaves of sorghum varieties with different levels of aphid resistance and found that the aphid resistance of sorghum is related to the leaf cell morphology, cell arrangement closeness, and blade thickness. A joint analysis of the transcriptome and metabolome was performed to obtain a large dataset related to sorghum response to aphid infestation. The results of the transcriptome–metabolome association analysis indicated that flavonoid biosynthesis plays an important role in the response of sorghum to aphids. To verify whether flavonoids affected sorghum resistance to aphids, we designed an exogenous spray experiment in which three concentrations (0, 0.1‰, and 0.2%) were selected for foliar application on the three sorghum varieties. The exogenous spraying of flavonoids showed that naringenin and genistein effectively increased the resistance of aphid-sensitive plants. The genes and metabolites identified in this study have provided new insights into the mechanisms underlying the response of sorghum to aphid infestation, and exogenous spraying of flavonoids may represent a potential approach for the biological control of sorghum aphids. However, the optimal application concentrations and mechanism of action in sorghum aphid resistance need to be further explored.

## Supplementary Information

Supplementary Material 1.

Supplementary Material 2.

Supplementary Material 3.

Supplementary Material 4.

Supplementary Material 5.

Supplementary Material 6.

Supplementary Material 7.

Supplementary Material 8.

Supplementary Material 9.

Supplementary Material 10.

## Data Availability

The dataset provided in this study has been uploaded to NCBI, The names of the repository/repositories and accession number(s) can be found below: BioProject, PRJNA1009505 (https://www.ncbi.nlm.nih.gov/sra/PRJNA1009505).
